# Mr. Soden, on Hydrophobia

**Published:** 1807-05

**Authors:** John Smith Soden

**Affiliations:** Member of the College of Surgeons, London. Coventry


### *To the Editors of the Medical and Physical Journal.*

GENTLEMEN,

OF all the calamities to which human nature is liable, hydrophobia is at once the most distressing and fatal. Pain and affliction of every other kind admit of alleviation, if not removal; but this disease always presents a scene of woe closed by death. Although much zeal and ability have been evinced by authors in commenting upon the nature of this direful malady, all attempts to cure it hitherto, have failed. Its mortality is undiminished; and few practitioners, in the present day, have confidence in any medicine, when once the poison has been absorbed. Fortunately, there is in our power, a mode of prevention, whose success is so well established, that a neglect of it is little less than criminal. The disease is an inoculated one; its virus is conveyed into the system by the same channel that small-pox and lues venerea are: and a removal of the saliva, or infecting fluid, by assiduous ablution, and extensive excision of the parts bitten, as certainly will prevent hydrophobia, as an early application of the same means to an inoculated arm would prevent variola. Caustics and cauteries are remedies of the same kind, but less secure; and alone ought never to be depended upon, if the knife can be employed. It is unnecessary to dwell upon other prophylacticss; these are the only efficacious plans yet suggested, and shortly, I hope, will supersede those futile nostrums by which the public so long has been deluded.

A few instances are upon record, in which this complaint is said to have been cured; but it behoves us to receive these accounts with caution, for if they be carefully examined, it will apppear that their authors have mistaken some some other disease for hydrophobia. Moderate scepticism ought always to be permitted in medical subjects; and liberal, but temperate discussion, will be objected to by none who are desirous to increase the knowledge, and extend the blessings of the healing art.

I have been led to these reflections by reading Mr. Hicks's communication in a late Journal. His case is interesting, but I think those who peruse it with attention, will not allow it to be an instance of genuine hydrophobia. It is well known, that a large majority of the dogs supposed to be mad, really are not so. In the present instance, no proof whatever is adduced of the madness of the animal at the time the patient was bitten. It is stated, that two men, who were in pursuit of the dog, intending to shoot him, said that he was mad; but, as neither his preceding nor subsequent history is given to us, their assertions cannot be deemed conclusive. The interval, between the infliction of the wound and the accession of the disease, was shorter than is usual in hydrophobia. More violenc was shewn early in this case, than ever is manifested in true rabies, except in the last stage. Hydrophobic patients, at the commencement, are timid, and easily managed; this man was frantic, and ungovernable. He does not appear to have been affected with ptyalism, or pria-pism, which are general concomitants of hydrophobia. Nothing is said of that strange appearance in the eyes, a mixture of wildness and timidity, which is peculiar to rabies canina.

The aversion to water, alone, is not sufficient to decide the nature of the complaint, as this symptom sometimes occurs, where not the least suspicion exists of the patient having been exposed to the hydrophobic virus. A gentleman, of considerable professional celebrity, informed me that he saw a case of tetanus, (in a young man who had wounded his finger by a splinter of wood), in which, hydrophobia was so predominant, that had he visited him without any previous knowledge of the origin of his disease, he should have attributed it to an injury from a rabid animal. The patient died. It would be easy to quote a number of similar instances, but the fact is so universally known, that it is unnecessary.

Mr. Hicks's patient no doubt was apprehensive of danger, from the time he was informed that the dog was mad; and, in a person strongly under the influence of terror, I can readily believe all the symptoms related might occur, without the intervention of a morbid poison. The case case was worth recording, and may be added to those which abound in the writings of medical authors, and tend to shew the power of imagination in aggravating disorders of the body: but, it has so little claim to be considered as genuine hydrophobia rabiosa, that a further refutation is needless.

The distress of the unhappy sufferers, in this dreadful malady, is inconceivable by those who have not witnessed it. There is sometimes, little or no difficulty in taking solids,* but the aversion to fluid is always so great as to preclude the possibility of swallowing any considerable quantity of food, or medicine in that form. The inability to introduce active remedies, in a liquid state, into the stomach, in the usual way, is, perhaps one cause of the inefficacy of every mode of treatment; but it appears to me, that this obstacle to success, might be removed by passing a flexible tube, something like an elastic gum catheter, down the œsophagus into the stomach. I do not know that convulsions would be excited by fluids conveyed into the stomach without immediate contact with the pharynx. The nourishment, or remedies, to be thus administered, should be carefully excluded from the patient's sight, and conducted into the tube with caution, which, I conceive, might be done by a syringe with a pipe well adapted to the aperture of the canula.

If an insurmountable impediment should resist the passage of the instrument through the pharynx; would it be justifiable to perform the operation of œsophagotomy, and pass a tube into the stomach from the wound?

I offer these remarks as hints for others to improve; and if they should possess any tendency to mitigate the horrors of a disease, whose malignity has baffled every effort that skill and ingenuity have devised for its relief, I shall sincerely rejoice.

I am, &c.

JOHN SMITH SODEN,

Member of the College of Surgeons, London.

*Coventry, March* 29, 1807.

To

* In a case, which I saw at the Birmingham Hospital, about ten years ago, the patient ate bread and meat within a few hours of his death.

